# Myometrial mechanoadaptation during pregnancy: implications for smooth muscle plasticity and remodelling

**DOI:** 10.1111/j.1582-4934.2008.00306.x

**Published:** 2008-03-17

**Authors:** X Wu, K G Morgan, C J Jones, R M Tribe, M J Taggart

**Affiliations:** a School of Clinical & Laboratory Sciences, University of ManchesterGreat Britain; b Department of Health Sciences, Boston UniversityMA, USA; c Division of Reproductive Health, Endocrinology & Development, King's CollegeLondon, Great Britain; d Institute of Cellular Medicine, School of Surgical and Reproductive Sciences, Newcastle UniversityGreat Britain

**Keywords:** mechanotransduction, smooth muscle remodelling, dense plaques, myometrium, smooth muscle plasticity

## Abstract

The smooth muscle of the uterus during pregnancy presents a unique circumstance of physiological mechanotransduction as the tissue remodels in response to stretches imposed by the growing foetus(es), yet the nature of the molecular and functional adaptations remain unresolved. We studied, in myometrium isolated from non-pregnant (NP) and pregnant mice, the active and passive length–tension curves by myography and the expression and activation by immunoblotting of focal adhesion-related proteins known in other systems to participate in mechanosensing and mechanotransduction. *In situ* uterine mass correlated with pup number and weight throughout pregnancy. *In vitro* myometrial active, and passive, length-tension curves shifted significantly to the right during pregnancy indicative of altered mechanosensitivity; at term, maximum active tension was generated following 3.94 ± 0.33-fold stretch beyond slack length compared to 1.91 ± 0.12-fold for NP mice. Moreover, mechanotransduction was altered during pregnancy as evidenced by the progressive increase in absolute force production at each optimal stretch. Pregnancy was concomitantly associated with an increased expression of the dense plaque-associated proteins FAK and paxillin, and elevated activation of FAK, paxillin, c-Src and extracellular signal-regulated kinase (ERK1/2) which reversed 1 day post-partum. Electron microscopy revealed close appositioning of neighbouring myometrial cells across a narrow extracellular cleft adjoining plasmalemmal dense plaques. Collectively, these results suggest a physiological basis of myometrial length adaptation, long known to be a property of many smooth muscles, whereupon plasmalemmal dense plaque proteins serve as molecular signalling and structural platforms contributing to functional (contractile) remodelling in response to chronic stretch.

## Introduction

Mechanical forces are important regulators of cell and organ physiology. Gene and protein expression, cell growth, migration and contractility [1–4] are strongly influenced by mechanical stretch in a variety of cell types, especially smooth muscle cells [5]. The sensitivity of smooth muscle to mechanical signals allows for rapid changes in muscle length and provides a physiological (and pathophysiological) mechanism by which hollow organs respond to volume changes [5–7] for example, in airway and vascular smooth muscle regulation of, respectively, alveoli oxygen tension and blood flow. *In vitro*, airway and vascular smooth muscle exhibits considerable plasticity in active and passive contractile properties in response to changing mechanical stimuli. This has been proposed to be mediated, in part, by alterations in the activation state of focal adhesion kinase (FAK) and paxillin which is dependent on actin filament polymerization [6, 8–11]. FAK and paxillin congregate in cellular focal adhesion sites rich in integrins, known as dense plaques in smooth muscle, and act as sensors/transducers connecting the extracellular matrix (ECM) to the cytoskeleton [2–5]. Mechanical forces are transduced into chemical signals; integrin activation results in autophosphoryla-tion of FAK, creating a high-affinity binding site for c-Src which facilitates downstream phosphorylation of paxillin which subsequently binds to the actin-linking protein vinculin [5]. The latter also interacts with talin, an important mediator in focal adhesion complex formation that binds directly to actin and β-integrins [12]. c-Src interaction with FAK and/or paxillin can also recruit additional signalling proteins and activate extracellular signal-regulated kinase (ERK1/2) [13–16].

Much less is understood about the nature of mechanoadaptive events in smooth muscle *in vivo*. A situation that presents a profound, yet entirely physiological, challenge of mechanical strain to a smooth muscle tissue *in vivo* is that of pregnancy and its influence upon uterine smooth muscle (myometrium) function. In pregnancy, the uterus has to respond to the unique physical challenge of changing mechanical strain due to uterine occupancy by the foetus(es), placenta(s) and amniotic fluid. The myometrium must differentially respond to these mechanical strains to allow for (a) appropriate growth and *in utero* survival of the foetus and (b) timely contractile effort at the end of gestation to safely expel the foetus(es) and placenta(s).

In rodents, evidence suggests that expression and/or activation of dense plaque proteins, such as α5 integrin, FAK, paxillin and ERK1/2, are elevated in late pregnancy and that acute stretch *in vitro* increased activation of focal adhesion-related protein [17–20]. However, a role for dense plaques in transducing mechanical strain in contracting uterus has been questioned [18]. Furthermore, the nature of any uterine smooth muscle mechanosensitisation with gestation and the relation of this to *in situ* uterine growth or dense body-related protein expression has not been addressed.

The present study focuses on the hypothesis that stretch of the uterus during pregnancy is an important signal acting *via* dense plaques for the induction of uterine growth and optimization of the contractile apparatus for efficient force production near term. Therefore, we assessed in non-pregnant (NP) (at different stages of the oestrous cycle) and pregnant mice (day 7–19 of gestation, term day 19): (*i*) markers of *in vivo* uterine growth; (*ii*) *in vitro* mechanoadaptation of myometrial contractility by measurement of the length-dependence of force production as well as the maximal force production at optimal length and *(iii)* the expression and activation of dense plaque-associated proteins. We show that significant alterations in the length–tension relationships, as well as adhesion plaque signalling, occur during pregnancy in the mouse. The results have implications for our understanding of myometrial functional adaptations during pregnancy, but are also relevant to our understanding of remodelling events occurring in response to mechanical strain in smooth muscle in general. Furthermore, the data from mice are especially relevant as setting a functional and quantitative baseline for transgenic mouse studies.

## Material and methods

### Materials

Monoclonal antisera to phospho-FAK (pTyr-397), phospho-paxillin (pTyr-118), paxillin, FAK and caveolin-1 were purchased from BD Transduction Laboratories (Oxford, UK). Monoclonal antisera to vinculin talin and α-actin were obtained from Sigma-Aldrich Company Ltd (Dorset UK). Rabbit polyclonal antisera raised against phospho-c-Src (pTyr 418) and c-Src were purchased from Biosource International (Camarillo, CA, USA) and Santa Cruz Biotechnology, Inc. (CA, USA), respectively. Rabbit polyclonal antisera raised against phospho-ERK1/2 and ERK1/2 were obtained from Cell Signaling, Inc. (MA, USA).

### Animals

Adult CD-1 mice were housed individually under standard environment conditions. The different stages of the oestrous cycle in NP mice were determined by histological examination of vaginal smears. Female virgin mice were mated with male CD-1 mice. Day 1 of gestation was designated as the day a vaginal plug was observed. Term was day 19.

### Tissue collection

Animals were killed by cervical dislocation according to UK Home Office national guidelines and myometrial samples were collected from (*i*) NP mice at different stages of the oestrous cycle (designated di-oestrous, pro-oestrous and oestrous); (*ii*) pregnant mice on gestational days 7 (D7) 13 (D13), 15 (D15), 17 (D17) and 19 (D19) and (*iii*) mice 1 day post-partum (1Dpp). Each uterine horn was quickly excised and weighed, then opened up along the mid-line. In pregnant mice, the placentas and foetuses in each horn were rapidly removed, the pups weighed and the empty horn re-weighed. Emptied uterine horn weight and pup weights per horn were averaged for each animal. Longitudinal myometrium strips were then isolated from the uterine tissue under a dissecting microscope and cleaned of any blood by rinsing in modified Krebs solution (154 mM NaCl, 5.4 mM KCl, 1.2 mM MgSO_4_, 1.6 mM CaCl_2_, 5.5 mM glucose, 10 mM 13-(N-Morpholino)-propanesulfonic acid (MOPS), pH 7.4). The myometrial tissues were immediately (*i*) snap frozen in liquid nitrogen and stored at -80°C for subsequent protein analyses, (*ii*) prepared for use in isometric contractile studies or *(iii)* prepared for electron microscopy.

### Measurement of length-tension and contractile properties of myometrium

Small strips of myometrium (4–5 mm length × 3 mm wide × 1 mm thick) were tied at each end with silk thread and mounted in a standard organ bath chamber (Linton Instruments, UK). One tissue end was tied to a fixed hook, the other to a TSD105A tension transducer (Linton Instruments, UK) linked to a micrometer allowing for manual stretch of the tissue in as little as 0.25 mm increments. The tissue bath contained physiological saline solution (PSS; 119 mM NaCl, 4.7 mM KCl, 2.4 mM MgSO_4_.7H_2_O, 25 mM NaHCO_3_, 1.18 mM KH_2_PO_4_, 0.03 mM K_2_ ethylenediaminetetraacetic acid (EDTA), 6.05 mM glucose, 1.6 mM CaCl_2_.2H_2_O, pH 7.4) at 37°C that was bubbled with a 95% air −5% CO_2_ mixture. Muscle strips were initially held at slack length (the length at which any further 0.25 mm incremental stretch of the tissue noticeably changed passive tension), equilibrated for 30 min. at this initial length and then stimulated with high K**^+^** solution (PSS with 60 mmM KCl substituted for NaCl) or 30 μm carbachol for 5 min. After washout and a further 10 min. equilibration in PSS, the tissues were stretched by 1 mm (approximately 1.20–1.25-fold beyond initial length). After 10 min. equilibration at this new length, the passive tension was noted just prior to the tissues again being stimulated with high K**^+^** solution or 30 μm carbachol for 5 min. and returned to PSS. The amplitude of any contraction, and the integrated contractile response, in response to the high K**^+^** or carbachol stimulus was noted. This procedure was repeated at several tissue lengths to allow construction of active and passive length–tension curves. For comparison of contractility between pregnancy and non-pregnancy, data are expressed as absolute force per cross sectional area (mN/mm^2^). Four mice for each stage of the oestrous cycle and each gestational age were used for these experiments.

### Western blot analysis

Frozen mouse myometrial samples were homogenized in modified RIPA lysis buffer (50 mM Tris-HCl [pH 7.4], 150 mM NaCl, 1% NP-40, 1 mM EDTA, 1 mM phenylmethylsulphonyl fluoride (PMSF), 1 mM Na_3_VO_4_, 1 mM NaF and protease inhibitor cocktail [Sigma Chemicals, St Louis, MO, USA]). Samples were centrifuged at 15,000 ×*g* at 4°C for 10 min. and the super-natants were collected. Protein concentrations were determined using Bio-Rad DC Protein Assay reagents. In one series of experiments, uterine samples from NP mice in pro-oestrus or oestrus were run on gels together with samples of mice in di-oestrus to allow for relative protein quantifications between different stages of the oestrous cycle. As the proteins of interest did not vary in expression during the oestrous cycle (see below), in all subsequent series' of experiments uterine samples from each selected gestation were run on one gel together with a sample from a NP mouse to allow for protein quantifications between gestational states relative to non-pregnancy. This process ensured identical treatment of samples from different conditions throughout the entire detection procedure.

Protein samples (50 μg/lane) were separated by SDS-PAGE, using poly-acrylamide (8–12%) gels, and electroblotted to 0.22 μm polyvinylidene difluoride (PVDF) membrane. The membranes were cut at appropriate molecular weights and blocked in 5% milk powder for 2 hrs at room temperature or overnight at 4°C (blocking solution was 5% bovine serum albumin (BSA) for detection of phosphoproteins). Primary antibodies were diluted in blocking solution and incubated with membranes for 2 hrs at room temperature or overnight at 4°C. Membranes were rinsed 3 × 15 min. in Tris-buffered saline (20 mM Tris base, 137 mM NaCl, pH 7.6) with 0.1% Tween-20 (TBST). Appropriate secondary antibodies, conjugated to horseradish peroxidase, were also diluted in blocking solution and incubated with membranes for 1 hr at room temperature. Membranes were washed 3 × 15 min. in TBST, then stained with enhanced chemiluminescent (ECL) substrate (Super Signal West Pico Chemiluminescent Substrate, Pierce, Rockford, IL, USA) and visualized on photographic film (Kodak, UK). Membranes stripped of originally bound primary antibody, were re-probed with other primary antibodies, incubated with secondary antibody and stained with ECL as above. Photographic films were densitometrically scanned and protein quantification was analysed by gel-pro analyser 4.5. Comparisons between conditions for any one protein of interest were performed as follows: all densitometric raw data values for any one protein of interest were made relative to α-actin from the very same membrane lane (see below). In addition, tissues of pregnant mice were compared to the values obtained for the same amount of NP homogenate (normalized to 1.0) on the same gel/membrane. Thus, a ratio of the amounts of each protein of interest (or of the phospho-specific signal of FAK, ERK1/2, c-Src or paxillin indicative of activated protein status), relative to a constant internal calibration of α-actin, was calculated for each gestational age *cf.* NP conditions. Similar methods of analyses have been applied in previous studies of myometrial protein expression by Western blotting [21–22].

### Electron microscopy

Dissected myometrial tissue from D19 mice were immediately immersed in fixative (2.5% gluteraldehyde in 0.1 M sodium cacodylate buffer pH 7.3) for 2 hrs at room temperature. The tissues were then washed several times in 0.1M sodium cacodylate buffer, to which 3 mM CaCl_2_ had been added, and stored at 4°C. Tissues were post-fixed in the dark (2 hrs, room temperature) in 1% osmium tetroxide in 0.1 M sodium cacodylate buffer. After a rinse in buffer, tissues were dehydrated in an ascending alcohol series, treated twice (15 min. each) with propylene oxide and left in a 1:1 mix of propylene oxide and Taab epoxy resin for 1 hr. Blocks were rotated overnight at 4°C in a mixture of 1:3 propylene oxide and epoxy resin followed by three changes (1 hr each) of fresh resin at 46°C before flat embedding in Beem capsules or rectangular moulds and polymerized at 60°C for 72 hrs. Ultrathin sections of transverse or longitudinal orientation to the longitudinal muscle were cut with a diamond knife, mounted on copper grids and stained with uranyl acetate and lead citrate. Specimens were examined in a Philips EM 301 or CM10 electron microscope at an accelerating voltage of 60 or 80 kV.

### Data analysis

Both active and passive tensions were analysed in relation to the length of the muscle preparations. A polynomial fit function (Originpro 7.0) was used to fit the active and passive tension data for each experimental dataset. The peak tension value for each condition was obtained from the fitted curve and taken as the maximal active tension. The active tensions at chosen stretches beyond the slack length were also calculated from the fitted curves and expressed as the percentage of the maximal active tension. All of the data were expressed as mean ± SD. *N*= number of mice/samples per stage of oestrous cycle or gestational age; *n*= number of times that the sample sets were electrophoresed and immunoblotted. The result of protein expressions by Western blot, myometrium length-tension properties and stimulated contractility data were analysed using One-way anova followed by a Dunnett test with respect to values in non-pregnancy. The correlation coefficient (*r*) between horn weights and pup weights was assessed by using a Pearson's correlation coefficient. Differences between individual groups were determined by a Student–Newman-Keuls test. *P* < 0.05 was considered statistically significant.

## Results

### Growth of Pup weights and uterine horn weights during pregnancy are significantly correlated

**Fig. 1:**
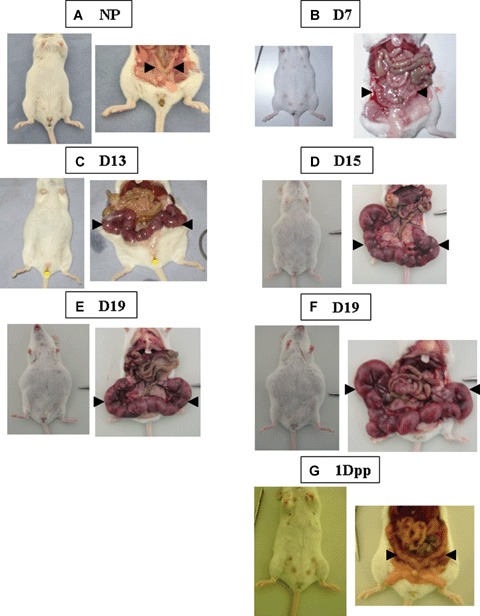
Uterine growth during pregnancy. Pictures of the growth of the abdominal area and, following dissection, of the exposed uterus (indicated by black arrowheads) between non-pregnancy (**A**, NP), D7 (**B**), D13 (**C**), D15 (**D**), D17 (**E**) and D19 (**F**) of pregnancy and 1Dpp (**G**).

**Fig. 2:**
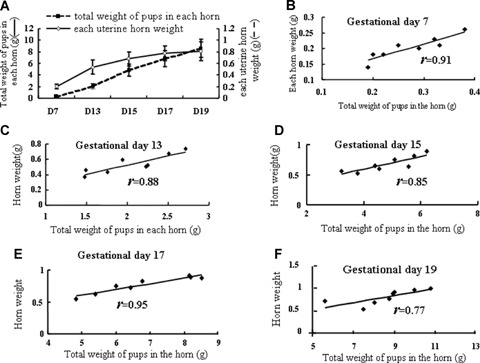
The relationship between uterine and pup weights during pregnancy. (**A**) Uterine and pup weight increased throughout gestation from D7-D19 (*N*= 4). The statistically significant correlations between pup weights and horn weights on each gestational day were *r*= 0.91, 0.88, 0.85, 0.95 and 0.77 on gestational days 7, 13, 15, 17 and 19 respectively (**B**-**F**).

Pregnancy was associated with dramatic increases in intrauterine volume (Fig. 1) which was partly reversed 1 day post-partum. We determined the uterine horn weight, and the weight of all pups in each horn, of each mouse (Fig. 2A and B). For each gestational day, the pup and uterine weight increased (Fig. 2A) with a statistically significant correlation; *r*^2^= 0.91, 0.88, 0.85, 0.95 and 0.77 on gestational days 7, 13, 15, 17 and 19 (*N*= 4, Fig. 2B–F) although, from Fig. 2A, it is evident that uterine weight has reached a plateau by day 17 even though the pups continue to grow in size until day 19.

### The length–tension properties of myometrium do not change during oestrous cycle but do change during pregnancy

**Fig. 3:**
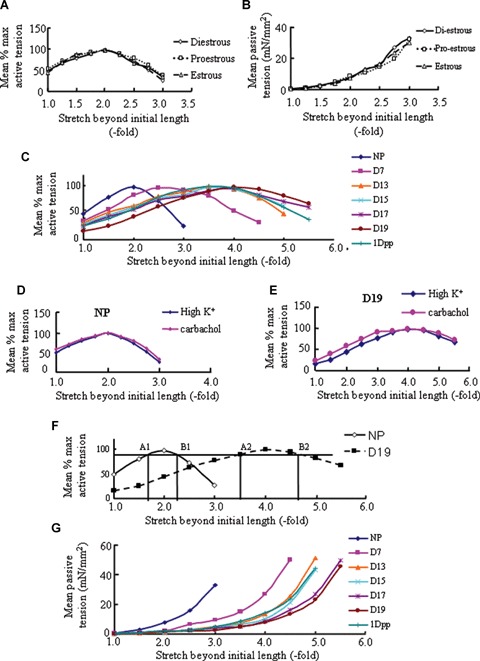
Length–tension properties of myometria from pregnant mice compared to non-pregnant mice. The active (**A**, high K**^+^** PSS stimulation) and passive (**B**) length–tension curves (*n*= 4) do not change significantly during the oestrous cycle of non-pregnant mice. In this and subsequent figures, the mean active tension depicted is normalized to the peak (100%) for each respective condition (in the case of **A**, di-oestrous, pro-oestrous, oestrous) and the stretch normalized to a slack length of 1.0. For clarity, the data are displayed without standard deviation error bars. (**C**) The active length-tension curves shifted progressively to the right during pregnancy compared to non-pregnancy (NP). (**D** and **E**) This right-ward shift, evidenced by comparing NP and D19, was irrespective of stimulation by High K**^+^** PSS or carbachol. (**F**) In myometrium of D19 mice, 90% of active tension could also be maintained over far greater stretches (A2-B2) than in NP tissue (A1–B1) upon high K**^+^** stimulation. (**G**) A rightward shift in the passive length–tension curves with pregnancy was also observed.

The active and passive length-tension relationships in the myometria of NP mice were unchanged during different stages of the oestrous cycle (Fig. 3A-B, *N*= 4). In contrast, pregnancy was associated with a dramatic rightward shift, and broadening of the plateau, of the active length-tension curves relative to the NP state. The prominent shift in mechanosensitivity was evident irrespective of whether the contractile agonist was carbachol (Fig. 3D-E) or high K**^+^** PSS (as detailed hereafter and in Fig. 3C). The rightward shift was partly reversed in tissue from 1Dpp mice. The altered mechanosensitivity associated with pregnancy was reflected in several measurements:

1. In the amplitude of contraction (relative to maximum) in the ascending portion of the active length-tension curves (Fig. 3C). Increasing muscle length 2-fold beyond slack length induced significantly less contraction at D19 (43.3 ± 6.4% of maximum), D17 (56.1 ± 7.5%), D15 (60.1 ± 9.9%) and D13 (63.3 ± 11.2%), but not D7 (82.9 ± 10.0%), than NP (95.5 ± 2.9%; Fig. 3C). Similarly, in order to achieve 70% maximum tension development, uterine smooth muscle strips from pregnant animals were stretched to a greater degree from slack length (D7 1.67 ± 0.14-, D13 2.26 ± 0.19-, D15 2.31 ± 0.24-, D17 2.41 ± 0.27- and D19 2.76 ± 0.25-fold respectively) compared to NP (1.30.1 ± 0.06-fold). The stretch required to allow development of 70% maximal tension on exposure to high K**^+^** PSS on D19 was significantly greater than on any other gestational days.
2. At the plateau phase of the length–tension curves. The stretch beyond slack length required to elicit maximum tension development was significantly greater from tissues of D7 (2.46 ± 0.21-fold), D13 (3.31 ± 0.33-fold), D15 (3.44 ± 0.40-fold), D17 (3.78 ± 0.45-fold) and D19 (3.94 ± 0.33-fold) compared to NP (1.91.1 ± 0.12-fold). This is also apparent when considering the range of stretches over which 90% of active force production was maintained in myometrium of NP or D19. In NP, this occurred at stretches beyond slack length of between 1.74 ± 0.09-fold to 2.14 ± 0.10-fold (A1–B1, Fig. 3F). In D19, however, this had significantly increased to between 2.80 ± 0.17-fold and 3.55 ± 0.22-fold
3. In the descending portion of the length-tension curves. Here, significantly greater stretches beyond slack length were required of tissue from D19, D17, D15, D13 and D7 (5.41 ± 0.12-, 5.15 ± 0.30, 4.83 ± 0.08-, 4.66 ± 0.16- and 3.76 ± 0.09-fold respectively) before active tension was reduced to 70% of maximum compared to NP (2.55 ± 0.06-fold).

**Fig. 4:**
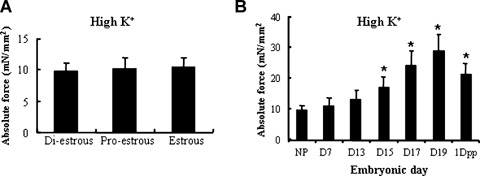
Effect of pregnancy on absolute active force production of myometria. (**A**) The absolute force production of myometrium from non-pregnant mice to high K**^+^** PSS did not vary with stages of the oestrous cycle. However, from D15 of pregnancy onwards there was a progressive enhancement of the force-producing capacity of myometrial tissue compared to non-pregnancy (**B**). Asterisks depicts *P* < 0.05.

In addition, when myometrial strips from pregnant mice were stretched to their respective optimal lengths for active tension development, there was a progressive enhancement of force production with advancing gestation (the force production at optimal length did not vary with the oestrous cycle; Fig. 4A). Contraction amplitudes to high K**^+^** PSS of tissues from D15 (17.04 ± 3.15 mN/mm^2^), D17 (24.26 ± 4.39 mN/mm^2^) and D19 (28.77 ± 5.48 mN/mm^2^), but not D7^−^ (11.29 ± 2.31 mN/mm^2^) or D13 (13.22 ± 2.84 mN/mm^2^), were significantly greater compared to NP (9.80 ± 1.21 mN/mm^2^; Fig. 4B). Similar data were obtained with agonist activation (data not shown). Absolute force development of myometrium from D19 (or D17) was also significantly greater than any other gestational day; furthermore, absolute force production 1Dpp had reduced to significantly less than that of D19.

The passive length-tension curves also showed a significant rightward shift with pregnancy (Fig. 3G). That is with pregnancy, progressively greater stretch had to be applied to the muscle to obtain the same passive tension as that seen in NP. This is evidenced by the observation that when the myometrial strips at each gestational day were stretched to their respective optimal lengths for active force production then the absolute passive tensions of tissues from D19 (7.5 ± 0.9 mN/mm^2^), D17 (8.5 ± 2.0 mN/mm^2^), D15 (6.7 ± 1.3 mN/mm^2^), D13 (7.2 ± 1.4 mN/mm^2^) or D7 (6.5 ± 1.5 mN/mm^2^) were not significantly different from that of NP (7.7 ± 1.2 mN/mm^2^).

### Myometrial α-actin protein expression does not change with oestrous cycle or pregnancy

**Fig. 5:**
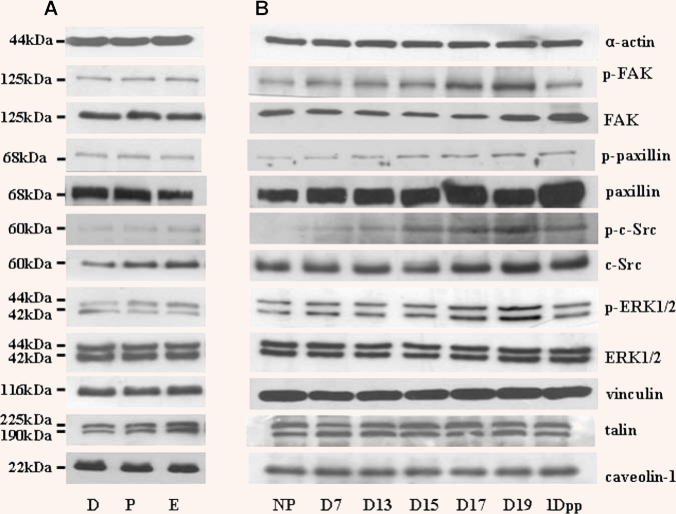
Effects of the oestrous cycle and pregnancy on the expressions of dense plaque-related proteins in mouse myometrium. Representative Western blots illustrating the expression of phospho-FAK, FAK, phospho-paxillin, paxillin, phospho-c-Src, c-Src, phospho-ERK1/2, ERK1/2, vinculin, talin and caveolin in myometrial tissue homogenates from non-pregnant (D = di-oestrous, p = pro-oestrous, e = oestrous), pregnant (D7-D19) and 1 day post-partum mice.

The proportion of α-actin expressed in myometrial tissue homogenates did not change during the oestrous cycle of NP mice (Fig. 5). The proportions of α-actin in pro-oestrus and oestrus mice were respectively 1.10 ± 0.12-fold and 1.06 ± 0.12-fold of that in di-oestrus mice (*N*= 4, *n*= 16). Thus, this enabled a comparison of other proteins of interest during the oestrous cycle to be made with α-actin probed on the very same blots. Additionally, the proportion of α-actin expressed in tissue homogenates did not change between NP mice and pregnant mice at different gestational days (Fig. 5). On an average, the α-actin proportions in D7, D13, D15, D17, D19 and 1Dpp samples were, respectively, 0.94 ± 0.11-, 0.97 ± 0.06-, 0.96 ± 0.06-, 1.05 ± 0.08-, 1.10 ± 0.12-and 1.03 ± 0.08- fold of that in NP samples (*N*= 4, *n*= 16). Hence, this allowed a comparison of other proteins of interest throughout gestation to be made with α-actin. As such, α-actin served as a constant, internal comparator for each of the other seven proteins of interest and also for the activation status (as measured by phospho-specific antibodies) of four of these proteins.

### Changes in the expression and activation of dense plaque-related proteins in myometrium during pregnancy are protein-specific

**Fig. 6:**
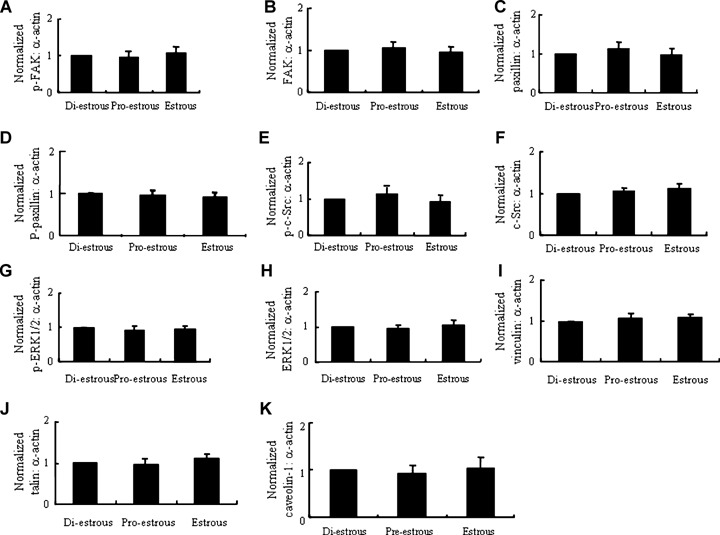
Mean data of dense plaque-related proteins during the oestrous cycle. Phosphorylation and expression of the proteins depicted in Fig. 5 was invariant during the oestrous cycle (*N*= 4).

**Fig. 7:**
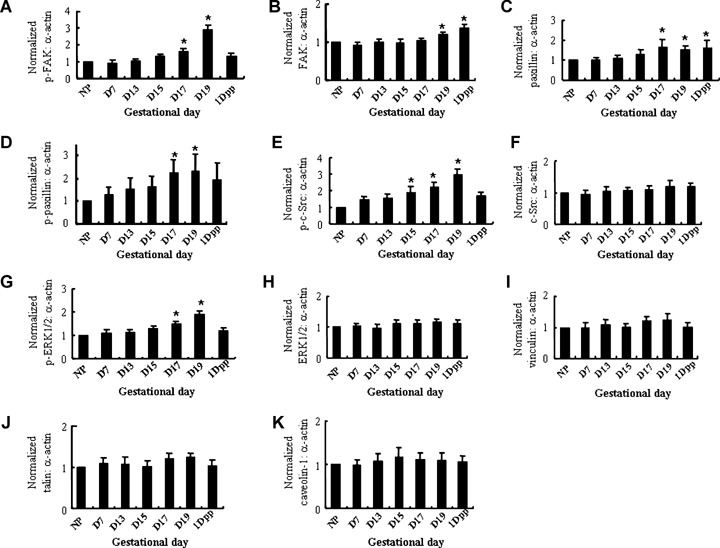
Mean data for the expression of dense plaque proteins in mouse myometri-um during pregnancy. Of the activation status of proteins examined in Fig. 5, phospho-FAK, phospho-paxillin, phospho-cSrc and phospho-ERK1/2 were each significantly increased on gestational day 17 and day 19 compared to non-pregnancy. Total FAK expression significantly increased on day 19, and total paxillin expression was significantly elevated on gestational days 17 and 19, compared to non-pregnancy. Asterisks depict *P* < 0.05.

The patterns of expression of putative dense plaque interacting proteins FAK, c-Src, paxillin, vinculin, talin, ERK1/2, as well as that of the non-dense plaque-related caveolae marker protein caveolin, did not change in myometrium during the oestrous cycle (Figs 5–6). In contrast, on D19 of pregnancy the expression of FAK relative to NP was significantly increased (1.18 ± 0.06-fold, *N*= 4) and this remained so 1Dpp (1.35 ± 0.10-fold). Paxillin expression was also elevated, compared to NP, on D17, D19 and 1Dpp (1.59 ± 0.31-, 1.88 ± 0.23- and 2.07 ± 0.12-fold respectively; Figs 5 and 7). All other protein expressions were invariant during pregnancy. The activity of FAK, as assessed by phospho-specific antibody recognition, was elevated during D17 (1.61 ± 0.15-fold, *N*= 4) and D19 (2.88 ± 0.31-fold) relative to NP. There were also increases in the activation of paxillin, c-Src and ERK1/2 on D17 (2.31 ± 0.71-fold, 1.94 ± 0.31-fold or 1.36 ± 0.17-fold respectively) and D19 (2.27 ± 0.54-fold, 2.49 ± 0.45 or 1.62 ± 0.18-fold) of pregnancy compared to NP (Figs 5 and 7). On 1Dpp, the activities of FAK(1.31 ± 0.18-fold compared to NP), ERK1/2 (1.21 ± 0.10-fold) and c-Src (1.68 ± 0.22-fold) were all significantly less than D19.

### Dense plaque ultrastructure in myometrium of pregnant mice

**Fig. 8:**
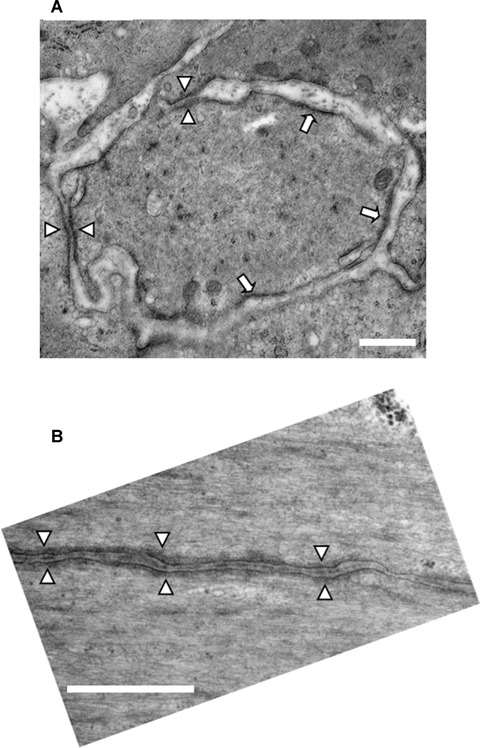
Electron microscopic examination of myometrial dense plaques. (**A**) Longitudinal myometri-um of D19 mice examined in cross-section reveals the plasma membrane to have prominent dense plaques (white arrows) interspersed with caveolae. Neighbouring cells are arranged in close proximity at regions of adjacent dense plaques (white arrowheads). Scale bar 500 nm. (**B**) In longitudinal section, myofilaments run roughly parallel to the long axis of the cell, meeting sarcolemmal dense plaques of adjacent cells (white arrowheads) at a shallow angle. Scale bar 500 nm.

The prominent appearance of plasmalemmal dense plaques in longitudinal myometrium from D19 mice was evidenced by electron microscopic examination of the tissue (Fig. 8). In cross-section, the dense plaques were interspersed with plasma membranous areas of caveolae. Moreover, neighbouring cells came into close proximity with one another at regions rich in dense plaques that were separated by a narrow (50 nm or less) cleft filled of ECM (Fig. 8A). In longitudinal section, the intracellular myofilaments were observed to run approximately parallel to the sarcolemma and dense plaques with the latter persisting for plasmalemmal lengths of ∼1.5 μm. Anchorage of filaments to the plasmalemmal dense plaques occurred, therefore, at a shallow angle (Fig. 8B).

## Discussion

During mouse pregnancy, the distending forces of growing pups and placentas result in a progressive increase in mouse uterine size. It is well known that mechanical stretch can induce uterine cell hypertrophy and hyperplasia [23–26] and here we show that pregnancy is also associated with a profound shift in mechanosensitivity of myometrial contractility. This is concomitant with an increased expression and activation of focal adhesion-related proteins after day 15 of pregnancy. We define mechanosensitivity as a shift in the length-sensitivity of contraction as distinct from mechanotransduction which is the cellular functional response, in this case force of contraction, at a particular mechanical stretch. Mechanotransduction at the optimal length for active contraction was also increased with pregnancy.

Pregnancy was associated with a progressive rightward shift in the myometrial active length–tension relationship such that, close to term (D19), the optimal length for tension development, and the range of lengths over which 90% of active tension could be produced, were approximately double that of NP tissue. This phenomenon of altered mechanosensitivity as a result of *in vivo* physiological circumstances resembles that observed in airway smooth muscle *in vitro*: pre-loading of tissues for 24 hrs at lengths less than optimal resulted in subsequent length–tension profiles that were sensitized relative to tissues preloaded at longer lengths [8]. These latter observations of historical stretch influencing subsequent length-dependence of contraction have highlighted the problematic issue of comparing ‘optimal lengths' between different studies, tissues and/or protocols [27]. Thus, for the purposes of our study, myometrial tissues extracted from NP or pregnant mice were treated with exactly the same experimental protocol. This ensured that the observed shifts in the length–tension curves were indeed related to the muscles history of stretch—in this case, that due to pregnancy *in vivo*. The shift in length–tension profiles seen in the present study of myometrium due to *in vivo* stretch associated with pregnancy are remarkably similar in nature to the above *in vitro* stretch experiments on airway smooth muscle, suggesting that analogous mechanisms may be at play. Amongst those thought to play a role in airway smooth muscle plasticity are: (*i*) a reduction in muscle stiffness or increased shortening velocity as the tissue is equilibrated at longer lengths compared to shorter lengths [6, 9]; (*ii*) dynamic myofila-ment reorganization with contractile stimulation and stretch [5, 9, 28] and (*iii*) increased activation of focal adhesion-signalling proteins with stretch [5]. In comparison, pregnancy has been associated with changes in the shortening velocity of permeabilized myometrium [29] (although contrasted with [30]), myometrial contractility is sensitive to inhibitors of actin filament polymerization [11] and, here, we report pregnancy to be associated with elevations of FAK and paxillin expression as well as altered activation of FAK, paxillin, c-SRC and ERK1/2 (see below). All of this suggests that cytoskeletal and myofilament remodelling contributes to the altered mechanosensitivity with pregnancy.

If the mechanisms of myometrial remodelling are indeed akin to that suggested *in vitro* in airway smooth muscle, then the shift in length-sensitivity with pregnancy could arise from an increased number of myofilaments registered in series [6, 9]. However, in airway smooth muscle, the altered mechanosensitivity is generally not associated with a change in the maximal force output [6, 8]. This contrasts with our study in which the myometrial shift in mechanosensitivity is also accompanied by a change in mechan-otransduction; that is, there is a progressive increase in the force-producing capacity of myometrium at the optimal lengths for each respective gestational day. This can partly be explained by the cell volume; unlike in the *in vitro* length-loading experiments on airway smooth muscle [6], the myometrial cell volume is not constant with pregnancy and, as α-actin content as a proportion of total protein remains constant, the cell hypertrophy may be associated with increased myofilament number. The relative proportions of filament arrangements in series or parallel relative to non-pregnancy could then determine the extent of shifts in both mechanosensitivity (length-dependence) and mechanotransduc-tion (force output).

In addition, there are at least two distinct mechanisms by which the uterus responds to the increasing volume during pregnancy [25, 26]. In rats, the first half of gestation involves myometrial cell hyper-plasia followed by cell hypertrophy in mid-gestation [26]. Towards term, however, (days 17 onwards in rat) there is no further hypertrophy [26]. If this is also true for mouse uterus, then from gestational day 17–19, when we observed continued foetal growth but a slowing of uterine weight gain, the adaptive mechanosensitive response may no longer be to alter the length-dependence of contraction but, rather, to increase the force producing capacity in preparation for the final initiation of labouring contractions.

**Fig. 9:**
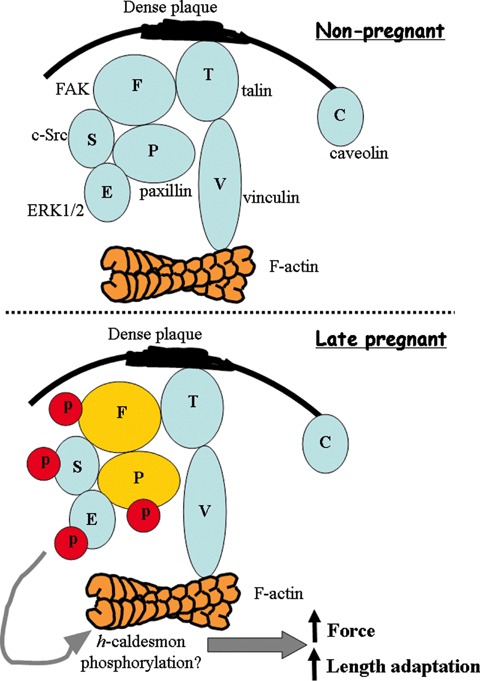
Schematic description of possible dense plaque-associated signalling in myometrium. Indicated are possible interactions between dense plaque-associated signalling molecules in myometrium of non-pregnant and late pregnant mice. Diagram constructed, in part, from information of [16, 19, 20, 45, 46]. Blue shading = no change in expression, yellow = increased expression in pregnancy, red = increased phosphorylation in pregnancy. C = caveolin, T = talin, F = Focal adhesion kinase (FAK), S = c-Src, P = paxillin, V = vinculin, E = ERK1/2. Although many non-muscle studies suggest that caveolin can also interact with proteins such as c-Src and ERK1/2, the distinct separate positioning of caveolae and dense plaques in smooth muscle (Fig. 8) suggest that if this occurs in myometrium then it is likely to be a as a result of distinct pools of signalling molecules (or as a result of stimuli distinct from gestational growth). One possible outcome of the increased activation (phosphorylation) of FAK, c-Src, paxillin and ERK1/2 may be a release of inhibitory actions of the thin filament-associated protein *h*-caldesmon [19, 20].

Dense plaques are thought to be regions of plasmalemma involved in mechanosensing and/or mechanotransduction. As such, we sought to examine if there was a correlation between the gestational-dependent *in vitro* mechanoadaptation and *in situ* expression and activation of putative dense plaque-associated proteins. As summarized in Fig. 9, phospho-FAK, phospho-paxillin, phospho-c-Src and phospho-ERK1/2 significantly increased especially on days 17 and 19 of gestation. The very similar patterns of these activated proteins imply that they are important signalling proteins in mechanoresponsive pathways.

The expression and activation of several integrin- and dense plaque-associated proteins may be under the dual control of endocrine and mechanical influences [17–18, 31]. *For the former*, several dense plaque-associated proteins may be responsive to progesterone withdrawal (and relative oestrogen increase) in rats although the steroidal sensitivity of similar proteins in mice is uncertain. An influential previous study reported that the activities of rat myometrial FAK and paxillin, but not c-Src nor ERK1/2, were reduced in late pregnancy [18]. The implication of the study in that aspects of dense plaque signalling may be down-regulated during labouring uterine contractile effort presents a conundrum yet to be fully resolved. A number of subsequent publications have tended to support a gestational-dependent increased expression of myometrial proteins that are thought to be associated with dense plaques (albeit activity is another matter) [17, 31–34]. Additionally, in other (non-vascular as well as vascular) smooth muscles, *in vitro* isometric contraction with exogenous stimulants activates dense plaque-associated proteins [5, 10] and expression of non-phosphorylatable paxillin mutants inhibits force development [35]. These data suggest that smooth muscle contractile effort may be associated with activation rather than inhibition of dense plaque-related signalling events.

*For the latter*, it is pertinent that *in vitro* mechanical stretch of a variety of cell types, including myometrium, directly results in activation of dense plaque/focal adhesion-related signalling proteins [36–39]. From our study, the progressive rightward shifts in length–tension curves *in vitro*, together with changes in expression and/or activation of dense plaque-related proteins, resembled the positive correlations between pup and uterine weight *in situ*; activation of dense plaque-associated proteins continued towards gesta-tional days 17–19 when uterine weight gain slowed compared to foetal weight gain. This lends further substance to the notion that mechanical stretch acting upon dense plaques, at least partly, was responsible for the molecular adaptations and functional remodelling.

Although not investigated in this study, there is an increasing realization that relative protein expression changes may be differentially regulated in circular and longitudinal smooth muscle layers during gestation [17, 32–34] although the relevance of these have yet to be extrapolated. Note, it is difficult to dissect from late pregnant mice pure myometrial circular muscle free of endometrium and longitudinal muscle that remains viable for *in vitro* mechanical studies. In the longitudinally-oriented strips reported here, the predominant mechanical influence of course resides in the longitudinal fibres. Nonetheless, one can imagine that the mechanoadaptations monitored *in vitro* as a result of *in utero* changes may also be prevelant in the circular muscle. Of particular note, is the finding that the cytoskeletal thin filament protein γ-actin expression increases predominantly in circular myometrium in mid- to late pregnancy of rats [32]. This remains to be ascertained in mice as, in our hands, the commercially available antibodies to γ-actin do not distinguish this protein from other actin isoforms in smooth muscle homogenates of 1-dimensional western blots.

The activation of ERK1/2 may have a pivotal role in the mechanoadaptive process as it is known that protein tyrosine kinase activity was required in the signal transduction pathway leading to ERK1/2 activation in stretched myometrial smooth muscle cells [38] and the tyrosine kinase-dependence may be associated with Src activity as it is a common upstream mediator of mechanical stress-induced ERK activation [36]. In our study the similar gestational-dependent activation patterns of phospho-c-Src, phosphor-paxillin and phospho-ERK1/2 are consistent with the possibility that the latter is a downstream effector of dense plaque-mediated mechanotransduction in myometrium. The influence of acute *in vitro* stretch of myometrium on dense plaque-associated protein activation will be a useful examination of this notion and preliminary experiments in this regard are supportive [20, 40]. In epithelial cells, growth factor-mediated Src activation of paxillin 118Y phosphorylation serves to recruit inactive ERK1/2 to bind to paxillin and subsequently effect downstream FAK activation [16]. It remains necessary to unravel with similar clarity the precise sequence of dense plaque-associated protein-protein interactions and signalling activations in the myometrium during gestation and labour onset (Fig. 9). Importantly, pregnancy and labour-induced ERK1/2 activation has been associated with phosphorylation (and presumed inhibition) of the thin filament-associated protein caldesmon, the expression of which is known to change during mouse pregnancy [22], and pharmacological inhibition of ERK1/2 activation delays parturition in an animal model of preterm labour [19–20]. Fig. 9 therefore suggests that *h-*caldesmon may be one potential myofilament effector protein of the upstream dense plaque-associated signalling pathways.

Pregnancy is associated with considerable alterations in myometrial ECM components, including in rat mRNA and/or protein of microfibrillar/adhesion proteins including type IV collagen, lamin, fibronectin—the ligand for α5β1 integrins—as does α5 integrin [17, 31, 33, 41–42], the nature of which may influence the profound rightward shifts in myometrial passive length–tension curves with pregnancy. It is noteworthy that the peak active force production of myometrial tissue from each gestational day occurred at approximately similar levels of passive tension. Thus, the passive elasticity/plasticity of the tissue is likely to be a predictive determining factor of the amount of active force-producing capacity of the tissue. Microfibrils of appearance indicative of fibronectin have been suggested to localize to the basement membrane of other smooth muscles [43] and, here, we report ultrastructural evidence to indicate that dense plaques of neighbouring myometrial cells are closely apposed (perhaps even joined) across a narrow cleft of ECM. These findings suggest that ECM-plasmalemmal dense plaque regions may be sites of structural linkage between myometrial cells as has been reported in tracheal smooth muscle [44]. Herein, may lay the clue to one physiological impact of dense plaque-associated protein activation with advancing gestation: to provide a structural and biochemical framework to co-ordinate contractions across myometrial cell bundles at term. Moreover, the broad plateau of the active length–tension curves of myometrium in late pregnancy, together with enhanced active force production per muscle stretch, will enable uterine tone to be maintained over the wide range of muscle shortenings and lengthenings likely to arise with periodic labouring contractions. In this way, each of these phenomena is likely to contribute to the optimization of a mechanical syncytium for labouring contractions [17].
